# Predictors of adequate physical activity within a multiethnic polycystic ovary syndrome patient population: a cross-sectional assessment

**DOI:** 10.1186/s12905-021-01257-w

**Published:** 2021-03-17

**Authors:** David Huang, Eleni Jaswa, Chia-Ning Kao, Molly Quinn, Marcelle Cedars, Heather Huddleston

**Affiliations:** 1 Department of Obstetrics, Gynecology and Reproductive Sciences, University of California San Francisco, 480 16th Street, 10th Floor, Box 0132, San Francisco, CA 94158 USA; 2 Division of Reproductive Endocrinology and Infertility, Department of Obstetrics, Gynecology and Reproductive Sciences, University of California San Francisco, San Francisco, CA USA; 3 Division of Reproductive Endocrinology and Infertility, Department of Obstetrics and Gynecology, University of California Los Angeles, Los Angeles, CA USA

**Keywords:** PCOS, Exercise, Race, Ethnicity, Health disparities

## Abstract

**Background:**

Physical activity is a cornerstone for treatment of women with polycystic ovary syndrome (PCOS), but there are limited data on their exercise behaviors. A previous study identified PCOS patients of non-White ethnicity to be at higher risk for inadequate physical activity. Further data is needed to identify groups that would benefit from additional counseling in achieving adequate physical activity (APA). Therefore, this study examined correlates of APA within a multiethnic PCOS patient population.

**Methods:**

Cross-sectional assessment of exercise behaviors within a multiethnic PCOS patient population was performed using the International Physical Activity Questionnaire (IPAQ). Kruskal–Wallis test was used to compare metabolic equivalents from physical activity among racial/ethnic groups. APA was defined as at least 150 min of moderate-intensity, or 75 min of vigorous-intensity, or an equivalent combination of moderate- and vigorous-intensity activity per week. Logistic regression analyses were performed to identify correlates of APA.

**Results:**

Four hundred and sixty-five women of various racial/ethnic backgrounds were included in analysis: 62% (n = 287) self-identified as White, 15% (n = 71) as Hispanic, 11% (n = 52) as East/Southeast Asian, 7% (n = 32) as South Asian, and 5% (n = 23) as Black/African American. Significant differences were observed in metabolic equivalents (METs) from vigorous-intensity and total (moderate plus vigorous-intensity) exercise across racial/ethnic groups (*p* < 0.01); South Asian patients had the lowest metabolic expenditure in moderate-intensity, vigorous-intensity, and total exercise. Overall prevalence of APA was 66%; South Asian patients exhibited the lowest prevalence (46.9%). Ethnicity was a predictor for APA when controlled for age (*p* = 0.01); this finding was attenuated in logistic regression models that also controlled for age and body mass index (*p* = 0.05) as well as education level and parity (*p* = 0.16).

**Conclusions:**

South Asian patients with PCOS exhibited the lowest metabolic expenditure and frequency of APA in our cohort. Differences in frequency of APA across racial/ethnic groups appear to be influenced by anthropometric and sociodemographic factors. Our findings present an opportunity for women’s health providers to be cognizant and provide additional counseling regarding physical exercise to at-risk PCOS patients to improve their known higher risk for adverse long-term metabolic outcomes.

**Supplementary Information:**

The online version contains supplementary material available at 10.1186/s12905-021-01257-w.

## Background

Polycystic ovary syndrome (PCOS) is a common yet complex clinical entity that affects 6–10% of reproductive-aged women, and is characterized by ovulatory dysfunction, hyperandrogenism and polycystic ovaries [1]. Beyond anovulatory infertility, PCOS has important implications for general health as these patients are often affected by other conditions, such as depression [2], and have an increased cardiovascular risk due to associations with obesity [3], lipid abnormalities [4], insulin resistance [5] and high blood pressure [6].

Lifestyle modifications, especially physical exercise, have been proposed to improve metabolic profiles, reproductive trajectories, as well as depressive symptoms, for patients with PCOS [2, 7–9]. It is also well recognized that physical inactivity is closely linked with the cumulative burden from chronic diseases and contributes significantly to mortality worldwide [10]. In 2008, the Department of Health and Human Services (DHHS) published updated guidelines for physical activity intended for adult Americans [11]. According to these guidelines, adults should perform at least 150 min of moderate-intensity exercise per week, or 75 min of vigorous-intensity exercise per week, or an equivalent combination of moderate- and vigorous-intensity activity in order to achieve substantial health benefits [11]. While the optimal exercise regimen for PCOS patients has not yet been determined, it has been shown that PCOS patients who are physically active demonstrate superior metabolic health parameters, especially those who perform vigorous-intensity exercise [12].

Previous studies have shown that a significant portion of women with PCOS do not conduct adequate physical exercise, and that those of non-White ethnicity was associated with a higher risk of inadequate physical activity [2, 12]. As the United States population continues to become more ethnically and socially diverse, efforts to better understand contributors to health disparities are needed. The objectives of this study were (1) to quantify exercise behaviors by metabolic equivalents and (2) to identify predictors of adequate physical activity within a multiethnic PCOS patient population in order to direct efforts in counseling high risk patients to improve their overall health outcomes.

## Methods

### Study population

We performed a cross-sectional assessment of patients diagnosed with Rotterdam-PCOS at a multidisciplinary PCOS clinic [13]. Patients were either self-referred or referred by outside providers for assistance in diagnosing and/or in management of PCOS. Patients were recruited consecutively between 2006 and 2019 to participate in a research cohort at the University of California, San Francisco. Cohort participants consented to systematic clinical and questionnaire-based data collection. Institutional Review Board approval was obtained.

Diagnosis of PCOS was made according to the Rotterdam ESHRE/ASRM criteria [13]. Evidence of clinical hyperandrogenism was defined by the presence of hirsutism using the modified Ferriman-Gallwey score ≥ 8 and/or severe androgen-dependent acne. Biochemical hyperandrogenism was defined by elevations in serum androgen levels (free testosterone, total testosterone, dehydroepiandrosterone sulfate, and/or androstenedione) above the resulting laboratory’s reference range. Participants were instructed to abstain from hormonally active medications for at least one month prior to serum testing. Exclusion of other endocrine disorders, such as hyperprolactinemia, thyroid dysfunction, and late-onset congenital adrenal hyperplasia, was also performed. Ovarian morphology was assessed via endovaginal ultrasound by a Reproductive Endocrinology and Infertility specialist, and was defined to meet polycystic ovarian morphology by the presence of at least 1 ovary with a volume greater than 10 cm^3^ and/or with ≥ 12 follicles that measured between 2 and 9 mm in diameter.

### Data collection

Before the initial clinic visit, patients were asked to complete a series of self-administered questionnaires. Demographic information gathered from these questionnaires pertinent to this study included self-reported race/ethnicity, menstrual history, age, income range, educational attainment, parity status, and place of birth. Anthropometric data including body mass index (BMI) and waist circumference were collected at the initial clinic visit.

Exercise data were collected prior to the initial visit using the International Physical Activity Questionnaire (IPAQ), which has been extensively validated across 12 countries as an international tool to measure physical activity [14]. The IPAQ consists of 27 questions that ask participants to report the amount of moderate and vigorous physical activity performed in the past 7 days related to work, house chores, transportation, and recreation/sport/leisure-time physical activity. In this questionnaire, moderate physical activity refers to those that take moderate physical effort and make one breathe somewhat harder than normal. Vigorous physical activity refers to those that take hard physical effort and make one breathe much harder than normal. Patients were evaluated for adequate physical activity as published by the DHHS. Adequate physical activity (APA) was defined as at least 150 min of moderate-intensity, or 75 min of vigorous-intensity, or an equivalent combination of moderate- and vigorous-intensity activity per week [11].

Exercise data were also analyzed by metabolic expenditure according to the amount of physical activity reported on the IPAQ. Metabolic expenditure was determined using metabolic equivalents (METs) as the unit, in which one MET is defined as the amount of oxygen consumed while sitting at rest. Its use represents a simplified and standardized way to express the energy cost of physical activity as a multiple of the resting metabolic rate [15]. According to the IPAQ scoring protocol, each minute of moderate physical activity was assigned four METs and each minute of vigorous physical activity was assigned eight METs. The weekly metabolic equivalents from moderate physical activity, vigorous physical activity, and total (moderate plus vigorous) physical activity were determined for each patient [14].

### Data analysis

Statistical analyses were performed using SAS v9.4, 32-bit edition. For sample size calculation, we performed a post-hoc power analysis. Given our population of 465 patients and our approximately 2:1 ratio of White to non-White subjects, we had adequate power to detect a difference of 15 percentage points in likelihood of achieving APA. Age and anthropometric data are reported by their median and 25th/75th percentile values. Baseline characteristics and demographic data were compared across racial/ethnic groups using Kruskal–Wallis test, Fisher’s exact test, or chi-square test as appropriate. Metabolic expenditure from moderate-intensity exercise, vigorous-intensity exercise, and total (moderate plus vigorous-intensity) exercise among ethnic groups was analyzed using Kruskal–Wallis test. Logistic regression was used to identify whether ethnicity, income level, educational attainment, parity status, and place of birth (U.S. or non-U.S. born) were potentially relevant predictors for adequate physical activity. All testing was performed at the 0.05 level of significance.

## Results

### Demographic and anthropometric data

A total of 465 women with PCOS completed the IPAQ and were included in these analyses. Characteristics of the study population are given in Table 1. Within the study population, 62% (n = 287) self-identified as White, 15% (n = 71) as Hispanic, 11% (n = 52) as East/Southeast (SE) Asian, 7% (n = 32) as South Asian (SA), and 5% (n = 23) as Black/African American (AA).

**Table 1:** Baseline patient characteristics

	White (n = 287)	Hispanic (n = 71)	East/SE Asian (n = 52)	South Asian (n = 32)	Black /AA (n = 23)	*p* value†
Age (year)*	28.3 (24.7, 32.1)	29.2 (23.4, 33.3)	29.0 (23.8, 33.7)	26.2 (23.5, 31.6)	32.0 (28.4, 36.6)	0.02
BMI*	26.0 (22.5, 32.9)	31.7 (27.9, 37.3)	25.7 (21.7, 31.5)	25.0 (22.0, 28.3)	36.2 (28.2, 40.7)	< 0.01
Waist Circumference (cm)*	81.3 (71.1, 96.5)	94.0 (81.3, 102.9)	81.3 (68.6, 91.0)	78.7 (68.6, 94.0)	101.6 (81.3, 117.0)	< 0.01
Percentage with income ≥ $75 k/year	53.4% (n = 139)	29.0% (n = 18)	51.0% (n = 25)	61.5% (n = 16)	47.8% (n = 11)	< 0.01
Percentage obtaining a college degree	78.7% (n = 210)	55.4% (n = 36)	69.4% (n = 34)	92.6% (n = 25)	57.1% (n = 12)	< 0.01
Percentage who are nulliparous	92.6% (n = 214)	85.5% (n = 53)	86.1% (n = 31)	90.9% (n = 20)	59.1% (n = 13)	< 0.01
Percentage who are U.S.-born	88.9% (n = 208)	75.4% (n = 43)	45.7% (n = 21)	47.8% (n = 11)	100% (n = 18)	< 0.01

^*^Data presented in median (25th percentile, 75th percentile)

^†^Kruskal–Wallis, chi-squared, or Fisher’s exact test used as appropriate

As shown in Table 1, the patient population was noted to have differences in baseline age, body mass index (BMI), and waist circumference. Further, the patient population also had differences in socioeconomic factors including income, level of education, parous status, and percentage who are U.S.-born. Pairwise comparison of baseline characteristics between racial/ethnic groups showed that the Black/AA cohort was significantly older compared to the White and South Asian cohorts. Comparison between the Black/AA and Hispanic cohorts did not show significant differences in BMI and waist circumference; however, these two cohorts both had significantly higher BMI and waist circumference when compared to the White, East/SE Asian, and South Asian cohorts (Additional file 1: Table A).

### Differences in exercise behaviors among racial/ethnic groups

We first quantified exercise behaviors of each group by metabolic equivalents (METs) and compared METs from moderate-intensity exercise, vigorous-intensity exercise, and total (moderate plus vigorous-intensity) exercise across ethnic groups (Table 2). We showed that METs from vigorous-intensity exercise and total exercise were significantly different across racial/ethnic groups (*p* < 0.01 and < 0.01, respectively). However, we did not observe significant differences in METs from moderate-intensity exercise across racial/ethnic groups. Of note, South Asian patients consistently showed the lowest metabolic expenditure among ethnic groups from moderate-intensity exercise, vigorous-intensity exercise, and total exercise. Unadjusted pairwise comparisons for the MET outcome suggested that the statistically significant differences for vigorous-intensity and total exercise were driven by differences between the South Asian and White cohorts (Additional file 1: Table B).

**Table 2:** Distribution of METs from moderate-intensity exercise, vigorous-intensity exercise, and total exercise among racial/ethnic groups

	Racial/ethnic group	N	Median (25th percentile, 75th percentile)		*p* value*
METs from moderate-intensity exercise	White	274	240	(0, 600)	0.08
	Hispanic	70	120	(0, 400)	
	East/SE Asian	50	290	(0, 720)	
	South Asian	30	0	(0, 240)	
	Black/AA	23	0	(0, 480)	
METs from vigorous-intensity exercise	White	276	960	(0, 1740)	< 0.01
	Hispanic	70	264	(0, 1440)	
	East/SE Asian	49	960	(0, 1440)	
	South Asian	31	0	(0, 960)	
	Black/AA	20	720	(0, 1800)	
METs from total exercise	White	287	1200	(480, 2400)	< 0.01
	Hispanic	71	720	(0, 1920)	
	East/SE Asian	52	960	(320, 2160)	
	South Asian	32	480	(0, 1200)	
	Black/AA	23	960	(0, 2400)	

*p values determined using Kruskal–Wallis test

### Predictors of adequate physical activity

We next evaluated exercise behavior using a threshold approach (adequate versus inadequate) by applying the DHHS guidelines [11]. Using a logistic regression model, controlled for age and BMI, we evaluated whether the following socio-demographic factors predicted APA: ethnicity (White, Hispanic, East/SE Asian, South Asian, or Black/AA), income level (dichotomized to either earning ≥ $75,000/year or not), education level (obtaining a college degree or not), parity (nulliparous or not), and place of birth (U.S.-born or not).

We found that there was a statistically significant difference in achieving APA among different racial/ethnic groups when controlled for age (*p* = 0.01, Table 3) and a trend towards statistical significance when both age and BMI are controlled (*p* = 0.05). Of note, South Asian patients exhibited the lowest frequency of APA. White ethnicity overall is associated with a higher likelihood of achieving APA compared to non-White ethnicity (*p* < 0.01, Table 4). We also found statistically significant differences in achieving APA by parity status and educational level (Tables 3, 4). Nulliparous patients were more likely to achieve APA compared to parous patients. Patients who have a college degree were more likely to achieve APA compared to those who do not. On the contrary, income level and being U.S.-born were not predictors of APA.

**Table 3:** Frequency of adequate physical activity by race/ethnicity, parous status, education level, income, and place of birth

Variable	Frequency of adequate physical activity	p value (adjusted for age)*	p value (adjusted for age and BMI)*
White	71.1% (n = 204)	0.01	0.05
Hispanic	54.9% (n = 39)		
East/SE Asian	67.3% (n = 35)		
South Asian	46.9% (n = 15)		
Black/AA	56.5% (n = 13)		
Nulliparous	64.1% (n = 255)	0.02	0.04
Parous	46.6% (n = 27)		
College graduate	69.2% (n = 255)	< 0.01	0.01
Not college graduate	55.1% (n = 86)		
Income ≥ $75 k/year	66.9% (n = 161)	0.27	0.50
Income < $75 k/year	62.9% (n = 171)		
U.S.-born	65.7% (n = 248)	0.45	0.16
Non U.S.-born	61.5% (n = 56)		

*p values determined by logistic regression

**Table 4:** Odds ratios of achieving adequate physical activity by race/ethnicity, parous status, education level, income, and place of birth

	Odds ratio (95% CI)	p value
White compared to non-White	1.83 (1.24–2.71)	< 0.01
Nulliparous compared to parous	1.59 (1.09–2.33)	0.02
College graduate compared to not	1.56 (1.21–2.01)	< 0.01
Earning ≥ $75 k/year compared to not	1.14 (0.91–1.43)	0.26
U.S.-born compared to not U.S.-born	1.12 (0.84–1.50)	0.44

We then sought to understand factors that contribute to differences in exercise behaviors across PCOS patients of different racial/ethnic groups. We performed multivariate logistic regression analyses to evaluate the relationship between race/ethnicity and APA, controlling for factors in addition to age and BMI, such as level of education and parous status. While the association of ethnicity and APA were significant when controlled for age alone (*p* = 0.01), this significance was slightly attenuated in multivariate logistic models that controlled for age and BMI (*p* = 0.05), as well as for age, BMI, education level, and parity status (*p* = 0.16; Additional file 1: Table C).

## Discussion

In the U.S., there is an emphasis on recognizing and addressing health disparities among Americans given our increasingly diverse population. In the field of obstetrics and gynecology, racial/ethnic disparities are well recognized, such as a higher risk of maternal mortality in Black women [16, 17] and worse IVF outcomes in patients on non-White ethnicity [18, 19]. Highlighting and understanding health disparities is an important first step to address contributing factors, which can subsequently be used to translate into improvement of health outcomes for at-risk groups. We sought to characterize exercise behaviors of women with PCOS from various racial/ethnic backgrounds, and to identify risk factors for inadequate physical activity within this patient group. We found that ethnicity, education level, and parity status are predictors of exercise behavior. Importantly, we identified South Asian PCOS patients specifically as a high-risk group for low metabolic expenditure and rates of APA. Women with PCOS who did not obtain a college degree and those who are parous also exhibited lower rates of APA.

To further elucidate the differences in frequency of APA seen among racial/ethnic groups, we performed logistic regression analyses controlling for multiple factors. We showed that the differences seen across ethnic groups are influenced by BMI and education level. As shown in Additional file 1: Table C, the relationship between ethnicity and APA was attenuated after both age and BMI were controlled (*p* = 0.05); this effect appeared to be mediated by differences in BMI (*p* = 0.02). After controlling for age, BMI, education level, and parity, ethnicity itself did not appear to significantly predict for adequate physical activity; this finding appeared to be driven by education level (*p* < 0.05). These findings suggest that sociodemographic and anthropometric factors play an important role in explaining disparities in exercise adequacy across racial/ethnic groups.

Overall, our findings suggest that South Asian PCOS patients as a group demonstrate less physical activity, both from a threshold perspective as well as by absolute amount of metabolic expenditure. This highlights South Asian patients with PCOS as a group that is at high risk for worse health outcomes given the association of PCOS and sedentary lifestyle with adverse metabolic parameters. Specifically, vigorous-intensity activity has been associated with improved metabolic parameters in PCOS patients and was also performed the least by South Asian patients in our study cohort [12]. Our findings supplement previous studies that assessed cardiovascular risk factors among Asian Americans compared to non-Hispanic Whites in the U.S. and found that Asian Indians demonstrate significantly less physical activity when compared to Whites in general [20]. A prior assessment focusing on metabolic profiles in PCOS patients also noted a high prevalence of metabolic syndrome in Indian women [21]. South Asian Americans also exhibit a significantly higher prevalence of type 2 diabetes mellitus than non-Hispanic Whites, Blacks, and Hispanics [22, 23]. Therefore, our findings emphasize the importance of addressing physical exercise with South Asian PCOS patients, as it is such a cornerstone for treatment of PCOS [7–9] and improvement in cardiovascular health [24].

We postulate that barriers to physical activity seen among South Asian PCOS patients are different from other ethnic minority groups in the U.S., as South Asian patients had the lowest mean BMI, highest proportion of achieving a college degree, and a high percentage being of nulliparous status in our study. A possible explanation is that social and cultural norms within the South Asian American community differ in their approach to promoting physical activity. For instance, a prior community-based study interviewing South Asian women regarding their perspectives on physical activity found that there was a notion in which physical activity is unnecessary if one is “skinny”, and a general lack of awareness for the benefits associated with exercise was observed [25]. Further, family disapproval was found to be a major barrier in this study, as South Asian women in the study were told that time should be spent on cooking and house chores, instead of on physical activity [25]. Gender-norms related to modesty are also of concern in some South Asian women, noting that the presence of men in facilities and the pressure for culturally appropriate gym attire were barriers to physical activity in this study [25]. Further research is needed to understand the unique challenges faced by South Asian PCOS patients in obtaining adequate physical exercise.

To our knowledge, this is the only observational study that describes exercise behaviors of U.S. PCOS patients with a specific focus on racial/ethnic variation. We recognize that our study has several limitations. Given the nature of a cross-sectional assessment, it is hard to ascertain whether the reported exercise behaviors represent the true exercise habits of these patients over a longitudinal period. Further, given the use of questionnaires for our data collection, it is possible that the patient’s self-reported ethnicity could be inaccurate or does not perfectly fit within the categories provided. The exercise data gathered from questionnaires may also introduce recall bias and potential inaccuracies. However, as compared to being interviewed directly by a researcher in person, the use of a survey potentially reduces the patient’s pressure to report an exercise amount that is not representative of their true behavior. We were also unable to capture which patients had previously received exercise counseling, potentially contributing to the differences seen between cohorts. Further, as these patients are all motivated in obtaining help with their PCOS condition, as evidenced by their presentation to the specialty clinic, our findings may be overestimated compared to the general PCOS population. The exercise trends of our Bay Area-specific patient population may also not be applicable to the general PCOS population. Lastly, while this is the largest observational study of exercise trends in women with PCOS, we also acknowledge that our findings are drawn upon relatively small sample sizes in certain racial/ethnic subgroups, which may limit the power of the logistic analyses. The relative contributions of different variables for each racial/ethnic group could be investigated in a future study once a large number of individuals were enrolled for each group.

## Conclusions

Our study supplements the limited literature on baseline exercise behaviors in the PCOS patient population, and contributes to our nation’s efforts to address health disparities by evaluating exercise trends by race/ethnicity. South Asian patients with PCOS exhibited the lowest metabolic expenditure and frequency of APA in our cohort. We also propose that different racial/ethnic groups face different barriers to physical activity. Our findings present an opportunity for women’s health providers to be cognizant and provide additional counseling regarding physical exercise to at-risk PCOS patients to improve their known higher risk for adverse long-term metabolic outcomes. Continued recruitment and data collection from patients of various racial/ethnic groups are needed to further substantiate our findings and identify additional risk factors for inadequate physical activity. These continued efforts will help provide guidance on addressing physical inactivity and promote behaviors that improve health outcomes across a diverse patient population.

## Supplementary Information

**Additional file 1:** Supplementary Data.

## Data Availability

The datasets used and/or analyzed during the current study are available from the corresponding author on reasonable request.

## Abbreviations

PCOS: Polycystic ovary syndrome
DHHS: Department of Health and Human Services
IPAQ: International Physical Activity Questionnaire
APA: Adequate physical activity
MET: Metabolic equivalent
BMI: Body mass index
