# Supplementary material for: Predictors of adequate physical activity within a multiethnic polycystic ovary syndrome patient population: a cross-sectional assessment
Source: BMC Womens Health. 2021 Mar 17;21:108. doi: 10.1186/s12905-021-01257-w (PMC7972197; doi:10.1186/s12905-021-01257-w)
Supplement: Supplementary file 1 — Additional file 1: Supplementary Data. [file 12905_2021_1257_MOESM1_ESM.docx]

**SUPPLEMENTARY DATA:**

**Table A.** Pairwise comparison of age, BMI, and waist circumference between racial/ethnic groups

|  | **Racial/Ethnic groups compared** | **p value** |
| --- | --- | --- |
| **Age** | White vs. Black/AA | **0.01** |
|  | White vs. Hispanic | 1.00 |
|  | White vs. East/SE Asian | 1.00 |
|  | White vs. South Asian | 1.00 |
|  | Black/AA vs. Hispanic | 0.06 |
|  | Black/AA vs. East/SE Asian | 0.09 |
|  | Black/AA vs. South Asian | **<0.01** |
|  | Hispanic vs. East/SE Asian | 1.00 |
|  | Hispanic vs. South Asian | 1.00 |
|  | East/SE Asian vs. South Asian | 1.00 |
|  | | |
| **BMI** | White vs. Black/AA | **<0.01** |
|  | White vs. Hispanic | **<0.01** |
|  | White vs. East/SE Asian | 1.00 |
|  | White vs. South Asian | 1.00 |
|  | Black/AA vs. Hispanic | 1.00 |
|  | Black/AA vs. East/SE Asian | **<0.01** |
|  | Black/AA vs. South Asian | **<0.01** |
|  | Hispanic vs. East/SE Asian | **<0.01** |
|  | Hispanic vs. South Asian | **<0.01** |
|  | East/SE Asian vs. South Asian | 1.00 |
|  | | |
| Waist Circumference | White vs. Black/AA | **<0.01** |
|  | White vs. Hispanic | **<0.05** |
|  | White vs. East/SE Asian | 1.00 |
|  | White vs. South Asian | 1.00 |
|  | Black/AA vs. Hispanic | 1.00 |
|  | Black/AA vs. East/SE Asian | **<0.01** |
|  | Black/AA vs. South Asian | **<0.01** |
|  | Hispanic vs. East/SE Asian | **<0.01** |
|  | Hispanic vs. South Asian | **0.04** |
|  | East/SE Asian vs. South Asian | 1.00 |

**Table B.** Unadjusted pairwise comparison of METs from vigorous-intensity and total exercise between racial/ethnic groups

|  | **Racial/Ethnic groups compared** | **p value** |
| --- | --- | --- |
| **METs from**  **vigorous-intensity exercise** | White vs. Black/AA | 0.94 |
|  | White vs. Hispanic | 0.09 |
|  | White vs. East/SE Asian | 0.78 |
|  | White vs. South Asian | **0.02** |
|  | Black/AA vs. Hispanic | 0.99 |
|  | Black/AA vs. East/SE Asian | 1.00 |
|  | Black/AA vs. South Asian | 0.78 |
|  | Hispanic vs. East/SE Asian | 0.95 |
|  | Hispanic vs. South Asian | 0.87 |
|  | East/SE Asian vs. South Asian | 0.58 |
|  | | |
| **METs from**  **total exercise** | White vs. Black/AA | 0.91 |
|  | White vs. Hispanic | 0.11 |
|  | White vs. East/SE Asian | 0.92 |
|  | White vs. South Asian | **<0.01** |
|  | Black/AA vs. Hispanic | 0.99 |
|  | Black/AA vs. East/SE Asian | 0.99 |
|  | Black/AA vs. South Asian | 0.77 |
|  | Hispanic vs. East/SE Asian | 0.76 |
|  | Hispanic vs. South Asian | 0.77 |
|  | East/SE Asian vs. South Asian | 0.17 |

**Table C.** Logistic regression models evaluating race/ethnicity as a predictor for inadequate physical activity

|  | p-values | | | | |
| --- | --- | --- | --- | --- | --- |
|  | **Race/**  **Ethnicity** | **Age** | **BMI** | **Education Level** | **Parity** |
| **Model 1**: Controlling for age | 0.01 | 0.99 |  |  |  |
| **Model 2**: Controlling for age and BMI | 0.05 | 0.83 | 0.02 |  |  |
| **Model 3**: Controlling for age, BMI, education level, and parity | 0.16 | 0.74 | 0.06 | <0.05 | 0.10 |
